# A Planar Conformation and the Hydroxyl Groups in the B and C Rings Play a Pivotal Role in the Antioxidant Capacity of Quercetin and Quercetin Derivatives

**DOI:** 10.3390/molecules16119636

**Published:** 2011-11-21

**Authors:** Mohamed Moalin, Gino P. F. van Strijdonck, Maud Beckers, Geja J. Hagemen, Paul J. Borm, Aalt Bast, Guido R. M. M. Haenen

**Affiliations:** 1 Department of Toxicology, Maastricht University, 6200 MD Maastricht, The Netherlands; 2 Zuydlab, Hogeschool Zuyd, 6400 AN Heerlen, The Netherlands

**Keywords:** antioxidant capacity, flavonoids, methylation, quercetin, rutin

## Abstract

The polyphenol quercetin (**Q**) that has a high antioxidant capacity is a lead compound in the design of antioxidants. We investigated the possibility of modifying quercetin while retaining its antioxidant capacity as much as possible. To this end, the antioxidant capacities of **Q**, rutin, monohydroxyethyl rutinoside (monoHER) and a series of synthesized methylated **Q** derivatives were determined. The results confirm that the electron donating effect of the hydroxyl groups is essential. It was also found that the relatively planar structure of **Q** needs to be conserved. This planar conformation enables the distribution of the electron donating effect through the large conjugated π-system over the entire molecule. This is essential for the cooperation between the electron donating groups. Based on the activity of the compounds tested, it was concluded that structural modification at the 5 or 7 position is the most optimal to retain most of the antioxidant capacity of **Q**. This was confirmed by synthesizing and testing **Q5OMe** (**Q6**) and **Q7OMe** (**Q7**) that indeed displayed antioxidant capacities closest to **Q**.

## 1. Introduction

Flavonoids are polyphenolic compounds found in most plants. They account for a significant percentage of the chemical constituents of vegetables, fruits, and beverages such as tea and red wine. As a group they are implicated in the health benefits of fruit and vegetable consumption [1]. Isolated flavonoids have various potent biological effects, and they are used as nutraceuticals [2]. 2-(3,4-Dihydroxyphenyl)-3,5,7-trihydroxy-4H-1-benzopyran-4-one or quercetin (**Q**) is one of the most prominent dietary flavonoids [1,3,4]. It has a high antioxidant capacity, which is implicated in its health effects, and is a lead compound in the design of antioxidants. Chemical modification of quercetin has generated new derivatives with improved biological effect. One of these semi-synthetic antioxidants, *i.e.*, monohydroxyethyl rutinoside (monoHER), efficiently protects against the cardiotoxic effect of doxorubicin in mice [5], although it appeared to be less effective in humans [6]. The chemical modification explored in the present study was O-methylation, since this might improve the cellular uptake and metabolic stability of **Q** [7,8,9]. It is important to note that antioxidants such as **Q** can exert their beneficial effects through different mechanisms, e.g., scavenging radicals, metal chelation and interactions with enzymes [10]. This makes profiling antioxidants such as **Q** a daunting task with many pitfalls [11]. The essential feature of **Q** evaluated in the present study is its free radical scavenging. This was determined using the well-established 2,2'-azino-bis(3-ethylbenzthiazoline-6-sulphonic acid) radical (ABTS**^•^**) decolorization assay to determine the effect of O-conjugation on the scavenging capacity of **Q**. We investigated the possibility of modifying quercetin while retaining its antioxidant capacity as much as possible.

## 2. Synthesis

**Scheme 1:**
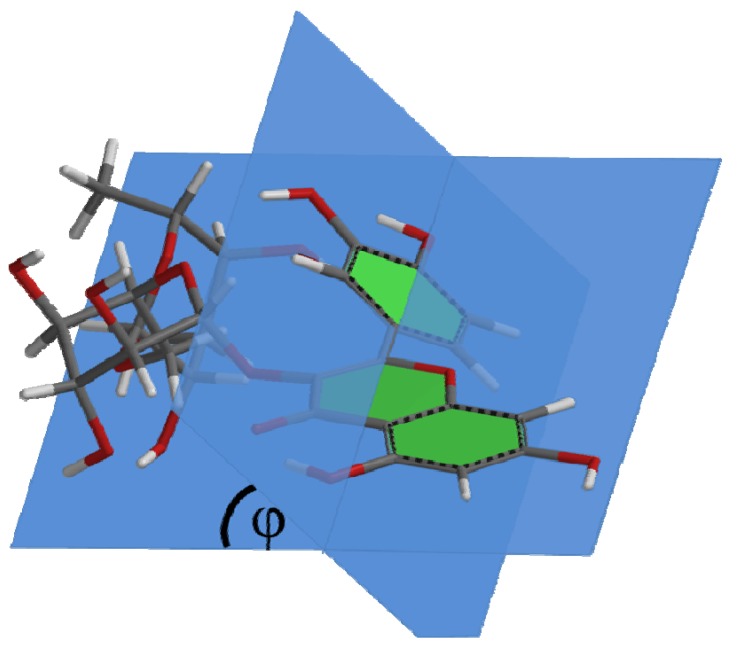
Synthesis of the methylated derivatives of quercetin.

The strategy used for the synthesis of the methylated derivatives of **Q** is adopted from Bouktaib *et al.* [12] and van Acker *et al. * [13]. The catechol moiety of **Q** was protected by the reaction with dichlorodiphenylmethane (Scheme 1) that lead to the formation of **1**. This reaction proceeds at temperatures above 160 °C. A major improvement appeared to be the addition of NMP, a solvent with a high boiling point, which increased the overall yield from 10 to 70%. The protected **Q** (**1**) was methylated with methyl iodide (MeI) and 1 equivalent (equiv.) K_2_CO_3_, followed by deprotection of the catechol group. Under these conditions both compounds **Q1** and **Q2** were formed and they could be readily separated by column chromatography on silica gel. Compounds **Q3** and **Q4** were synthesized by reacting **Q** with MeI and 3 eq of K_2_CO_3_. Both compounds could be readily separated by silica column chromatography. Methylation of **Q** with an excess of MeI and base gives the pentamethylated derivative **Q5**. **I3** Had already been already synthesized for an earlier study by demethylation of **Q5** with an excess of AlCl_3_. All the other synthesized compounds were made according to Bouktaib *et al.* and van Acker *et al.* with minimum adjustments. The chemical structures of the tested compounds are depicted in Table 1.

**Table 1:** The antioxidant capacities of quercetin (**Q**), isorhamnetin (**I1**), rutin (**R1**) and their derivatives. The capacity is expressed as the number of ABTS radicals (ABTS**^•^**) that is scavenged by one molecule of the tested compound. 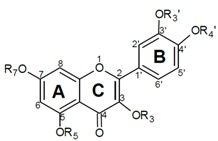

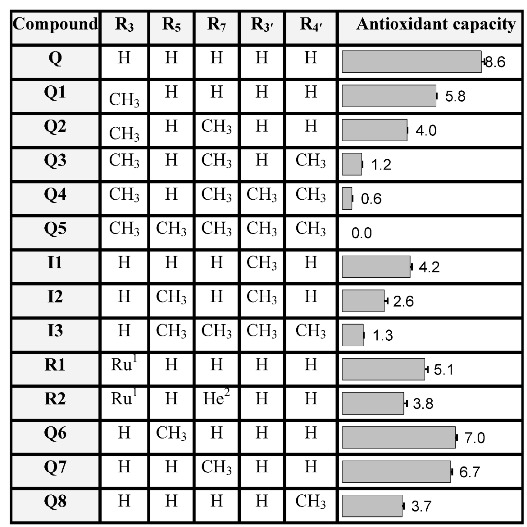

^1^ Ru is a rutinosyl group, a disaccharide consisting of glucose and rhamnose; ^2^ He is a hydroxyethyl group.

## 3. Results and Discussion

The antioxidant capacity was determined in a decolorization assay using the intensely colored 2,2'-azino-bis(3-ethylbenzthiazoline-6-sulphonic acid) radical (ABTS**^•^**), as described previously [14,15,16,17,18,19]. In our study, the capacity is expressed as the number of ABTS**^•^** that is scavenged by one molecule of the compound tested (Table 1). In the literature, the capacity of antioxidants is usually related to the capacity of the reference compound trolox and expressed as Trolox Equivalent Antioxidant Capacity (TEAC). These TEAC values have to be multiplied by 1.9, which is the value of Trolox in the assay, to compare TEAC data to our data. In literature, it is reported that **Q** can scavenge 5.9 [18], 8.4 [19], 9.0 [14] and 9.3 [17] ABTS**^•^**. The capacity observed for **Q** in the present study is 8.6. In addition, the reported values of rutin (**R1**) are 4.7 [14] and 5.2 [16], and we observed 5.1. This shows that the values found in the present study are within close proximity of most of the values reported previously.

In accordance with the literature data, **Q** has an extraordinarily high antioxidant capacity compared with simpler polyphenols, such as 4-methylcatechol (**MC**) that has a capacity of 2.5 in our assay. Compared to **MC**, **Q** contains a large conjugated π-system. This large conjugated π-system is one of the main reasons for the high antioxidant potential of **Q**.

Gradual methylation of the hydroxyl groups, from **Q** to **Q1**, **Q2**, **Q3**, **Q4** and **Q5** decreases the capacity (Table 1). Methylation of all the OH groups (**Q5**) reduces the capacity of **Q** to zero. This demonstrates that not only the size of the conjugated π-system determines the antioxidant potential; the free OH groups play a pivotal role in the antioxidant capacity as well.

It has been suggested that the antioxidant capacity of flavonoids is proportional to the number of OH groups present in the molecule [20]. However, several derivatives with the same number of OH groups display a significant difference in capacity. For example, the mono-methylated derivatives **Q1** and **I1**, both containing four OH groups, show a large difference of 1.6 in capacity (Table 1). In addition, there is also a substantial difference of 1.4 between the two di-methylated derivatives (**Q2** and **I2**) and of 0.7 between the two tetramethylated derivatives (**Q4** and **I3**). This confirms that the number of OH groups is not the primary factor in the antioxidant capacity of **Q** and its derivatives [21,22,23,24,25].

Apparently the contribution of each OH group to the antioxidant capacity is not equal. Methylation of the OH groups in the B-ring of **Q** reduced the antioxidant capacity the most, followed by methylation of the 3-OH group in the C-ring. Methylation of the 5 or 7 position in the A-ring reduced the antioxidant capacity the least.

The effect of OH groups on the antioxidant activity of flavones has previously been explained by their strong electron donating effect. Donating electrons to the conjugated π-system decreases the ionization potential and increases the energy of the highest occupied molecular orbital (HOMO) that is reflected by an increase in the antioxidant potential. The optimum stimulating effect of OH groups that are attached to **Q**, is found with an even number of C-atoms between these groups [21]. In addition, adjacent OH groups can also increase the antioxidant potential by stabilizing the flavone radical through intramolecular H-bonding interaction [24,25]. Consequently the OH groups in **Q** cooperate in the scavenging of radicals, and the efficiency of this cooperation depends on their relative position.

Previously, two pharmacophores have been postulated for **Q**; *i.e.*, the AC-ring and the B-ring [21,22,23]. The activity of various flavonoids indicated that the 3-OH group was the active centre in the AC-ring, and that its activity is positively influenced by the OH groups at the 5 and 7 position in the AC-ring [21]. In the present study, two tetramethylated derivatives of **Q**, *i.e.*, **Q4** and **I3**, were tested. **Q4 **has only one free OH group at the 5 position, and **I3** has only one free OH group at the 3 position. The capacity of **I3** (*i.e.*, 1.3) was twice that of **Q4** (*i.e.*, 0.6). This confirms that an OH group at the 3 position is indeed more important for the antioxidant activity than one at the 5 position.

The high activity of the B-ring can be explained by the strong electron-donating effect of its two adjacent phenolic OH groups and their intramolecular H-bonding interaction [21,24,25]. Actually, in our study methylation of the B-ring has the most profound effect as demonstrated by the relatively low capacity of **I1**. **I1** is methylated at the 3’-O position in the B-ring and its capacity is 4.4 lower than **Q**. For comparison, **Q1** is methylated at the 3-O position in the AC-ring and this leads to a decrease of 2.8. This confirms the importance of the OH groups in the B-ring for the antioxidant capacity of **Q**.

**Figure 1:**
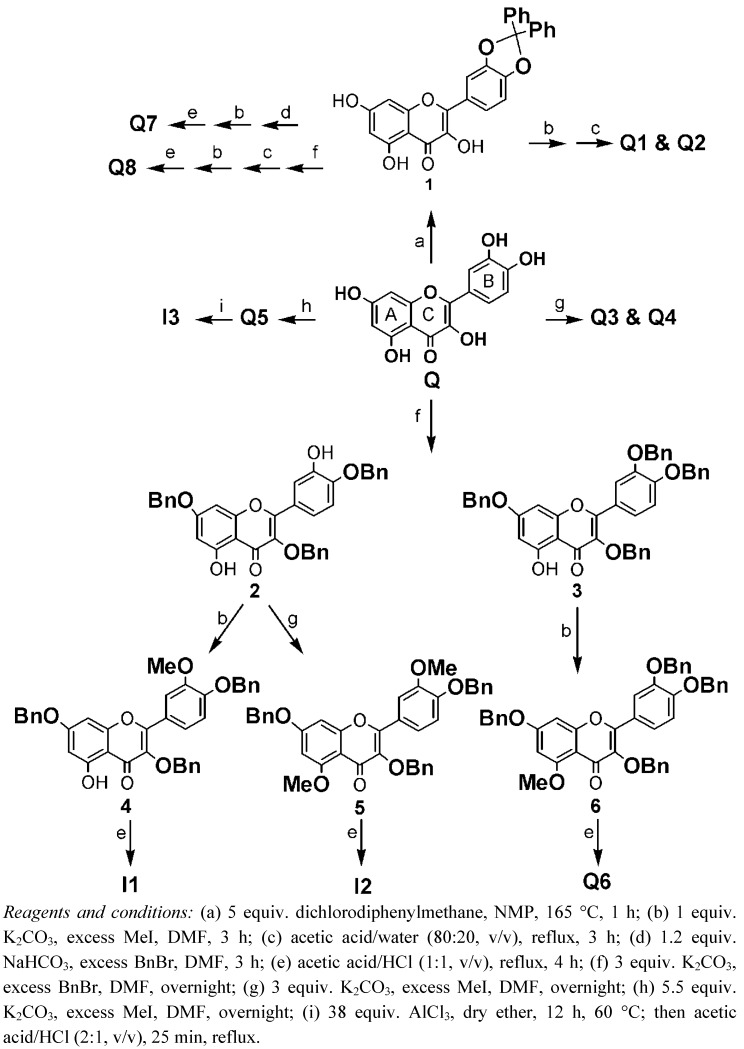
Three dimensional structure of rutin (**R1**), illustrating the dihedral angle (φ) of 38° between the plane of the AC-ring and that of the B-ring.

The difference in capacity (*i.e.*, 0.7) between **Q1** and **R1** (Table 1) indicates that other factors, besides the position and number of the OH groups, also influence the antioxidant capacity of **Q** derivatives. Both compounds are only substituted at the 3-O position. The difference is that **Q1** has a methyl group while **R1** has a rutinosyl group. To examine the effect of substitution at the 3-O position, quantum calculations on the equilibrium geometry of the molecules were performed [25]. The calculations point out that the bulky rutinosyl group at the 3-O position induces a dihedral angle (φ) of 38° between the plane of the AC-ring and that of the B-ring. The methyl group in **Q1** induces a dihedral of 20°, while in **Q** the dihedral is only 0.29° (Table 2). The relatively planar structure of **Q** allows the conjugated π-system of the AC-ring and that of the B-ring to efficiently interact. This interaction distributes the electron donating effect of the OH groups over the whole molecule, enabling the cooperation of electron donors in the different rings. Apparently, the pharmacophores that were postulated for **Q**, *i.e.*, the AC-ring and the B-ring, can cooperate. However, a bulky substituent at the 3 position, such as a rutinosyl group, forces the B-ring out of the plane of the AC-ring (Figure 1). This steric effect diminishes the efficiency of the large conjugated π-system found in **Q**, leading to a relatively low antioxidant capacity. In the design of derivatives of **Q** aimed at retaining most of the antioxidant activity, the relatively planar conformation of **Q** should be conserved.

**Table 2:** The antioxidant capacities of **Q**, **Q1** and **R1** *vs.* their dihedral angles (φ), the angle between the plane of the AC-ring and that of the B-ring.

Compound	Capacity	φ
Q	8.6	0.29°
Q1	5.8	20°
R1	5.1	38°

*In vivo*, methylation of the 3'-O or 4'-O in the B-ring by Catechol *O*-Methyl Transferase (COMT) is a major pathway. It has been suggested that these metabolites have a substantial contribution to the biological effects of **Q**, because their cellular uptake as well as their metabolic stability are superior to those of **Q** [8,9,26]. However, our data corroborate previous studies [9,27] that demonstrate the relatively low antioxidant capacity of these metabolites.

Our results show that semi-synthetic derivatives that are methylated at the 5 or 7 position display a higher antioxidant capacity than the *in vivo* metabolites. This was confirmed by synthesizing the 5 and 7 O-methylated quercetin derivatives (**Q6** and **Q7**). Moreover, 5 or 7 O-methylation of **Q** would not undermine the structural features that are important for metal chelation, *i.e.*, 3-hydroxy-4-keto conformation together with the 2,3-double bond and the catecholic B-ring [10,25]. To complete the series of monomethylated derivatives, we also synthesized **Q4′OMe** (**Q8**). In line with our theory, **Q8** appeared to be the least active, while **Q6** and **Q7** demonstrate the highest capacity of the O-conjugated derivatives of **Q**.

## 4. Experimental

### 4.1. Antioxidant Capacity

#### 4.1.1. Chemicals

All chemicals, synthesized or purchased, had a purity of at least 95% by HPLC. Rutin·3H_2_O, ABTS [2,2'-azinobis-(3-ethylbenzothiazoline-6-sulfonic acid)] and ABAP 2,2′-Azobis-(2-amidino-propane)·HCl) were purchased from Sigma-Aldrich and used as received. MonoHER [7-mono-*O*-(β-hydroxyethyl)-rutoside] was kindly provided by Novartis Consumer Health (Nyon, Switzerland). Spectrophotometric analysis was performed with a Varian Carry 50 spectrophotometer (Varian, Mulgrave, VIC, Australia).

#### 4.1.2. Scavenging of ABTS Radicals

All solutions were prepared fresh on the same day of the experiments. To generate the ABTS radicals (ABTS**^•^**), ABTS (20 mM) was incubated with ABAP (2.5 mM) in 145 mM sodium phosphate buffer (pH 7.4) for 30 min at 60 °C. During the experiments, the ABTS**^•^** stock solution was protected from light and kept on ice. The antioxidants were dissolved in DMSO to a concentration of 0.4 mM and further diluted in the phosphate buffer (pH 7.4) to a final concentration of 4 μM. In the actual measurement, antioxidant (500 μL of 4 μM solution) was mixed with ABTS**^•^** (500 μL of 60 μM solution) and incubated at 37 °C. After 5 min the absorbance at 734 nm was measured. The concentration of the ABTS**^•^** was determined by using its molecular extinction coefficient of 1.5 × 10^4^ M^−1^cm^−1^. Control experiments showed that DMSO and the phosphate buffer did not interfere with the assay. The antioxidant capacity is expressed as the number of ABTS**^•^** that has reacted with one molecule of the antioxidant within the 5 min incubation period. All measurements were performed, at least, in triplicate. Data are expressed as mean ± standard deviation.

### 4.2. Molecular Quantum Calculations

The density functional theory (DFT) using the B3LYP hybrid functional with the 6-31G* basis set in Spartan ’10 (Wavefunction, Irvine, CA, USA) was utilized for calculating the equilibrium geometry, and for determining the dihedrals (φ), the angles between the plane of the AC-ring and that of the B-ring, of quercetin (**Q**), rutin (**R1**) and quercetin-3-OMe (**Q1**).

### 4.3. Synthesis

#### 4.3.1. General

Quercetin·2H_2_O, dichlorodiphenylmethane, benzyl bromide, iodomethane, potassium carbonate, sodium bicarbonate, MgSO_4_ and silica-gel 60 A (0.060–0.200 mm) were purchased from Acros Organics and used as received. All solvents were obtained from Sigma-Aldrich and were of analytical grade. *N*-Methyl-2-pyrrolidone (NMP) and dimethylformamide (DMF) were distilled at reduced pressure before use. For the ^1^H-NMR data, chemical shifts are recorded in ppm downfield from tetramethylsilane. *J *values are given in Hz. Abbreviations used are s (singlet), d (doublet), dd (double doublet), t (triplet), b (broad) and bm (broad multiplet). Melting points (Mp) were measured on a differential scanning calorimetric apparatus (Mettler DSC 12E) and were not corrected.

#### 4.3.2. Protection of the Catechol Group of Quercetin. Synthesis of 2-(2,2-Diphenylbenzo [1,3]dioxol-5-yl)-3,5,7-trihydroxychromen-4-one (**1**)

To a solution of quercetin (2.0 g, 5.9 mmol) in NMP (60 mL), dichlorodiphenylmethane (5.8 mL, 30 mmol) was added. The solution was heated to 165 °C, stirred for 1 h and then cooled to room temperature (RT). The solution was diluted with EtOAc (150 mL), washed with 0.1 M HCl (100 mL) and saturated NaCl (aq.). The organic layer was dried over MgSO_4_, the drying agent was filtered and the filtrate was dried *in vacuo*. The crude product was purified by flash column chromatography using toluene/EtOAc (9:1, v/v) as eluent. The eluent was evaporated and compound **1** (2.0 g, 4.3 mmol, 70%) was thus isolated. ^1^H-NMR ([D3]MeCN, 300 MHz): 6.21 (d, *J *= 2.1 Hz, 1H, H_6_), 6.48 (d, *J *= 2.1 Hz, 1H, H_8_), 7.24 (dd, *J* = 8.7 Hz, 2.0 Hz, 1H, H_5′_), 7.46–7.61 (bm, 10H, diphenyl protons), 7.81 (d, *J* = 2.0 Hz, 1H, H_2′_), 7.84 (b, 1H, H_6′_), 12.39 (s, 1H, 5-OH).

#### 4.3.3. Synthesis of Quercetin-3-OMe (**Q1**) and Quercetin-3,7-OMe (**Q2**)

To a solution of **1** (0.60 g, 1.3 mmol) and potassium carbonate (182 mg, 1.3 mmol) in DMF (12 mL), an excess of iodomethane (0.20 mL, 3.2 mmol) was added. The reaction mixture (RM) was stirred at RT overnight for 3 h. The mixture was diluted with 0.1 M HCl (30 mL) and extracted with EtOAc (50 mL). The organic layer was washed with saturated NaCl (aq.) and dried over MgSO_4_. The drying agent was filtered and the filtrate was evaporated *in vacuo*. The resulting residue was refluxed in acetic acid/water (8:2, 100 mL) for 3 h and then cooled down to RT. The RM was diluted with water (100 mL) and extracted with EtOAC (200 mL). The organic layer was washed with saturated NaHCO_3_ (aq.) (100 mL), water (100 mL) and saturated NaCl (aq.) (100 mL). The organic layer was dried over MgSO_4_, the drying agent was filtered and the filtrate was dried *in vacuo*. The crude product was purified by flash column chromatography using toluene/EtOAc (10:3, v/v) to afford 100 mg of **Q1** (0.31 mmol, 24%) and 150 mg of **Q2** (0.45 mmol, 35%), after removal of the eluents *in vacuo*.

*Quercetin-3-OMe* (**Q1**). ^1^H-NMR ([D3]MeCN, 300 MHz): 3.79 (s, 3H, 3-OCH_3_), 6.20 (d, *J *= 2.1 Hz, 1H, H_6_), 6.42 (d, *J *= 1.8 Hz, 1H, H_8_), 6.91 (d, *J* = 8.4 Hz, 1H, H_5′_), 7.46 (dd, *J *= 8.4, 2.4 Hz, 1H, H_6′_), 7.56 (d, *J* = 2.4 Hz, 1H, H_2′_), 12.72 (s, 1H, 5-OH).

*Quercetin-3,7-OMe* (**Q2**). ^1^H-NMR ([D3]MeCN, 400 MHz): 3.81 (s, 3H, 3-OCH_3_), 3.87 (s, 3H, 7-OCH_3_), 6.38 (b, 1H, H_6_), 6.73 (b, 1H, H_8_), 7.12 (d, *J* = 8.8 Hz, 1H, H_5′_), 7.58 (b, 2H, H_2′_, H_6′_), 9.44 (s, 1H, 3′-OH), 12.65 (s, 1H, 5-OH).

#### 4.3.4. Synthesis of Quercetin-3,7,4′-OMe (**Q3**) and Quercetin-3,7,3′,4′-OMe (**Q4**)

To a solution of quercetin (0.25 g, 0.74 mmol) and potassium carbonate (0.30 g, 2.2 mmol) in DMF (6 mL), iodomethane (0.20 mL, 3.2 mmol) was added. The RM was stirred at RT overnight. The mixture was diluted with 0.2 M HCl (15 mL) and extracted with EtOAc (30 mL). The organic layer was washed with saturated NaCl (aq.) and dried over MgSO_4_. The drying agent was filtered and the filtrate was evaporated *in vacuo*. The resulting residue was purified by flash column chromatography using CHCl_3_/EtOAc (95:5, v/v) as eluent. Two products were isolated. The eluents were concentrated *in vacuo* and the products were precipitated from *iso*-hexane. The different precipitates were dried in vacuo to afford 0.13 g of **Q3** (0.39 mmol, 53%) and 46 mg of **Q4** (0.13 mmol, 17%).

*Quercetin-3,7,4′-OMe* (**Q3**). ^1^H-NMR ([D3]MeCN, 400 MHz): 3.81 (s, 3H, 3-OCH_3_), 3.87 (s, 6H, 4′-OCH_3_, 7-OCH_3_), 6.38 (d, *J *= 2.1 Hz, 1H, H_6_), 6.73 (d, *J *= 2.0 Hz, 1H, H_8_), 7.12 (d, *J* = 9.2 Hz, 1H, H_5′_), 7.58 (b, 2H, H_2′_, H_6′_), 9.44 (s, 1H, 3′-OH), 12.65 (s, 1H, 5-OH).

*Quercetin-3,7,3′,4′-OMe* (**Q4**). ^1^H-NMR (CDCl_3_, 300 MHz): 3.88 (s, 3H, 3′-OCH_3_), 3.89 (s, 3H, 3-OCH_3_), 3.98 (s, 3H, 4′-OCH_3_), 3.99 (s, 3H, 7-OCH_3_), 6.37 (d, *J *= 2.1 Hz, 1H, H_6_), 6.46 (d, *J *= 2.1 Hz, 1H, H_8_), 7.00 (d, *J* = 8.4 Hz, 1H, H_5′_), 7.70 (d, *J* = 1.8 Hz, 1H, H_2′_), 7.75 (dd, *J* = 8.4, 2.1 Hz, 1H, H_6′_), 12.65 (s, 1H, 5-OH).

#### 4.3.5. Synthesis of Quercetin-*penta*-OMe (**Q5**)

To a solution of quercetin (0.20 g, 0.59 mmol) and potassium carbonate (0.45 g, 3.3 mmol) in DMF (5 mL), iodomethane (0.40 mL, 6.4 mmol) was added. The RM was stirred at RT overnight. The mixture was diluted with 0.2 M HCl (15 mL) and extracted with EtOAc (30 mL). The organic layer was washed with saturated NaCl (aq.) and dried over MgSO_4_. The drying agent was filtered and the filtrate was evaporated *in vacuo*. The resulting residue was crystallized from EtOAc (1 mL) and then washed twice with cold EtOAc (0.5 mL). The crystals were dissolved in DCM, precipitated from *iso*-hexane and then dried *in vacuo* to afford **Q5** (0.10 g, 0.27 mmol, 46%).

*Quercetin-penta-OMe* (**Q5**). ^1^H-NMR (CDCl_3_, 300 MHz): 3.89 (s, 3H, 3′-OCH_3_), 3.92 (s, 3H, 3-OCH_3_), 3.97 (b, 9H, 5-OCH_3_, 7-OCH_3_, 4′-OCH_3_), 6.36 (d, *J *= 2.1 Hz, 1H, H_6_), 6.52 (d, *J *= 2.4 Hz, 1H, H_8_), 6.99 (d, *J* = 9 Hz, 1H, H_5′_), 7.70 (d, *J* = 1.8 Hz, 1H, H_2′_), 7.73 (b, 1H, H_6′_).

#### 4.3.6. Synthesis of Quercetin-3,7,4′-OBn (**2**) and Quercetin-3,7,3′,4′-OBn (**3**)

To a solution of quercetin (1.0 g, 3.0 mmol) and potassium carbonate (1.4 g, 10 mmol) in DMF (20 mL), benzyl bromide (2.2 mL, 18 mmol) was added. The RM was stirred at RT overnight. The mixture was diluted with 0.3 M HCl (60 mL) and extracted with EtOAc (100 mL). The organic layer was washed with saturated NaCl (aq.) and dried over MgSO_4_. The drying agent was filtered and the filtrate was evaporated *in vacuo*. The resulting residue was purified by flash column chromatography using CHCl_3_/EtOAc (95:5, v/v) to afford 0.80 g of **2** (1.4 mmol, 47%) and 0.66 g of **3** (1.0 mmol, 33%), after removal of the eluents *in vacuo*. *Quercetin-3,7,4′-OBn* (**2**): Mp 150.8 °C (150–152 °C), *Quercetin-3,7,3′,4′-OBn * (**3**): Mp 145.2 °C (140–142 °C) [12].

#### 4.3.7. Synthesis of Quercetin-3,7,4′-OBn-3′-OMe (**4**)

To a solution of **2** (0.20 g, 0.35 mmol) and potassium carbonate (50 mg, 0.36 mmol) in DMF (5 mL), an excess of iodomethane (0.10 mL, 1.6 mmol) was added. The RM was stirred at RT overnight. The mixture was diluted with 0.3 M HCl (15 mL) and extracted with EtOAc (30 mL). The organic layer was washed with saturated NaCl (aq.) and dried over MgSO_4_. The drying agent was filtered and the filtrate was evaporated *in vacuo*. The residue was purified by flash column chromatography using CHCl_3_/EtOAc (95:5, v/v) as eluent. The eluent was concentrated *in vacuo* and the residue was precipitated from methanol. The precipitate was dried *in vacuo* to afford 0.15 g (0.26 mmol, 74%) of *Quercetin-3,7,4′-OBn-3′-OMe * (**4**). Mp 141.8 °C (142–144 °C) [12].

#### 4.3.8. Synthesis of Quercetin-3,7,4′-OBn-5,3′-OMe (**5**)

To a solution of **2** (0.20 g, 0.35 mmol) and potassium carbonate (0.15 g, 1.1 mmol) in DMF (5 mL), an excess of iodomethane (0.10 mL, 1.6 mmol) was added. The RM was stirred at RT overnight. The mixture was diluted with 0.3 M HCl (10 mL) and extracted twice with EtOAc (30 mL). The combined organic layer was washed with saturated NaCl (aq.) and dried over MgSO_4_. The drying agent was filtered and the filtrate was evaporated *in vacuo*. The product was recrystallized from toluene and dried *in vacuo* to afford 0.13 g (0.22 mmol, 63%) of *Quercetin-3,7,4′-OBn-5,3′-OM*e (**5**). Mp 139.6 °C.

#### 4.3.9. Synthesis of Quercetin-3,7,3′,4′-OBn-5-OMe (**6**)

To a solution of **3** (0.20 g, 0.30 mmol) and potassium carbonate (50 mg, 0.36 mmol) in DMF (5 mL), an excess of iodomethane (0.10 mL, 1.6 mmol) was added. The RM was stirred at RT overnight. The mixture was diluted with 0.3 M HCl (15 mL) and extracted with EtOAc (30 mL). The organic layer was washed with saturated NaCl (aq.) and dried over MgSO_4_. The drying agent was filtered and the filtrate was evaporated *in vacuo*. The residue was purified by flash column chromatography using CHCl_3_/EtOAc (95:5, v/v) as eluent. The eluent was dried *in vacuo* to afford 0.18 g (0.27 mmol, 90%) of *Quercetin-3,7,3′,4′-OBn-5-OMe* (**6**). Mp 159.8 °C (156–158 °C) [12].

#### 4.3.10. Debenzylation of **4**, **5** and **6**: Synthesis of Quercetin-3′-OMe (**I1**), Quercetin-5,3′-OMe (**I2**) and Quercetin-5-OMe (**Q6**), respectively (see section Section 4.3.17 “General procedure for debenzylation”)

#### 4.3.11. Synthesis of 2-(2,2-Diphenylbenzo [1,3]dioxol-5-yl)-3-benzyloxy-5,7-dihydroxychromen-4-one (2*) and 2-(2,2-Diphenylbenzo [1,3]dioxol-5-yl)-3,7-dibenzyloxy-5-hydroxychromen-4-one (3*)

To a solution of **1** (0.60 g, 1.3 mmol) and sodium bicarbonate (0.13 g, 1.5 mmol) in DMF (12 mL), an excess of benzyl bromide (1.0 mL, 8.2 mmol) was added. The RM was stirred at RT overnight. The mixture was diluted with 0.1 M HCl (30 mL) and extracted with EtOAc (50 mL). The organic layer was washed with saturated NaCl (aq.) and dried over MgSO_4_. The drying agent was filtered and the filtrate was evaporated *in vacuo*. The resulting residue was purified by flash column chromatography using toluene/EtOAc (100:5, v/v) to afford 0.24 g (0.43 mmol, 33%) of *2-(2,2-Diphenylbenzo [1,3]dioxol-5-yl)-3-benzyloxy-5,7-dihydroxychromen-4-one* (2*) and 0.51 g (0.79 mmol, 61%) of *2-(2,2-Diphenylbenzo [1,3]dioxol-5-yl)-3,7-dibenzyloxy-5-hydroxychromen-4-one* (3*).

#### 4.3.12. Monomethylation of 2*: Synthesis of 2-(2,2-Diphenylbenzo [1,3]dioxol-5-yl)-3-benzyloxy-5-hydroxy-7-methoxychromen-4-one (4*)

To a solution of 2* (0.20 g, 0.36 mmol) and potassium carbonate (45 mg, 0.33 mmol) in DMF (5 mL), an excess of iodo methane (0.10 mL, 1.6 mmol) was added. The RM was stirred at RT overnight. The mixture was diluted with 0.1 M HCl (15 mL) and extracted with EtOAc (30 mL). The organic layer was washed with saturated NaCl (aq.) and dried over MgSO_4_. The drying agent was filtered and the filtrate was evaporated *in vacuo*. The resulting residue was purified by flash column chromatography using toluene/EtOAc (100:5, v/v) to afford 0.16 g (0.28 mmol, 78%) of 2-(2,2-Diphenylbenzo [1,3]dioxol-5-yl)-3-benzyloxy-5-hydroxy-7-methoxychromen-4-one (4*). Mp 134.8 °C (134–136 °C) [12].

#### 4.3.13. Debenzylation of 4*: Synthesis of Quercetin-7-OMe (**Q7**) (see Section 4.3.17 “General procedure for debenzylation”)

#### 4.3.14. Tribenzylation of Compound 1 Followed by Selective Deprotection of the Catechol Moiety: Synthesis of Quercetin-3,7-OBn (5*)

To a solution of **1** (0.60 g, 1.3 mmol) and potassium carbonate (0.55 g, 4.0 mmol) in DMF (12 mL), an excess of benzyl bromide (1.0 mL, 8.2 mmol) was added. The RM was stirred at RT overnight. The mixture was diluted with 0.1 M HCl (30 mL) and extracted with EtOAc (50 mL). The organic layer was washed with saturated NaCl (aq.) and dried over MgSO_4_. The drying agent was filtered and the filtrate was evaporated *in vacuo*. The resulting residue was refluxed in acetic acid/water (8:2, 100 mL) for 3 h and then cooled down to RT. The reaction mixture (RM) was diluted with water (100 mL) and extracted with of EtOAc (200 mL). The organic layer was washed with saturated NaHCO_3_ (aq.) (100 mL), water (100 mL) and saturated NaCl (aq.) (100 mL). The organic layer was dried over MgSO_4_, the drying agent was filtered and the filtrate was dried *in vacuo*. The crude product was purified by flash column chromatography using toluene/EtOAc (10:1, v/v) to afford 0.38 g (0.78 mmol, 60%) of *Quercetin-3,7-OBn* (5*), after removal of the eluents *in vacuo*. Mp 201.5 °C (202–204 °C) [12].

#### 4.3.15. Monomethylation of 5*: Synthesis of Compound Quercetin-3,7-OBn-4′-OMe (6*)

To a solution of 5* (0.28 g, 0.58 mmol) and potassium carbonate (75 mg, 0.54 mmol) in DMF (7 mL), an excess of iodomethane (0.10 mL, 1.6 mmol) was added. The RM was stirred at RT for 3 h. The mixture was diluted with 0.1 M HCl (15 mL) and extracted with EtOAc (30 mL). The organic layer was washed with saturated NaCl (aq.) and dried over MgSO_4_. The drying agent was filtered and the filtrate was evaporated *in vacuo*. The resulting residue was purified by flash column chromatography using toluene/EtOAc (100:5, v/v) to afford 0.20 g (0.40 mmol, 69%) of *Quercetin-3,7-OBn-4’-OMe* (6*). Mp 146.8 °C (144–146 °C) [12].

#### 4.3.16. Debenzylation of 6*: Synthesis of Quercetin-4′-OMe (**Q8**) (see section Section 4.3.17 “General procedure for debenzylation”)

#### 4.3.17. General Procedure for Debenzylation: Synthesis of Quercetin-3′-OMe (**I1**), Quercetin-5,3′-OMe (**I2**), Quercetin-5-OMe (**Q6**), Quercetin-7-OMe (**Q7**) and Quercetin-4′-OMe (**Q8**)

A suspension of 0.2 mmol of the benzylated product in HCl (36%, 10 mL) and glacial acid (10 mL) was refluxed for 4 h and then cooled down to RT. The reaction mixture (RM) was diluted with water (100 mL) and extracted with EtOAc (200 mL). The organic layer was washed with saturated NaHCO_3_ (aq.) (100 mL), water (100 mL) and saturated NaCl (aq.) (100 mL). The organic layer was dried over MgSO_4_, the drying agent was filtered and the filtrate was concentrated in vacuo. The products were precipitated from *iso*-hexane and the precipitates were dried *in vacuo*.

*Quercetin-3′-OMe* (**I1**). (60 mg, 0.19 mmol, 95%). ^1^H-NMR ([D6]DMSO, 300 MHz): 3.86 (s, 3H, 3′-OCH_3_), 6.21 (d, *J *= 2.1 Hz, 1H, H_6_), 6.49 (d, *J* = 2.1 Hz, 1H, H_8_), 6.95 (d, *J *= 8.4 Hz, 1H, H_5′_), 7.70 (dd, *J* = 8.4, 2.1 Hz, 1H, H_6′_), 7.77 (d, *J* = 2.1 Hz, 1H, H_2′_), 9.45 (b, 1H, 3-OH), 9.75 (b, 1H, 4′-OH), 10.79 (b, 1H, 7-OH), 12.48 (s, 1H, 5-OH).

*Quercetin-5,3′-OMe* (**I2**). (63 mg, 0.19 mmol, 95%). ^1^H-NMR ([D6]DMSO, 300 MHz): 3.84 (s, 3H, 3′-OCH_3_), 3.85 (s, 3H, 5-OCH_3_), 6.37 (d, *J *= 2.1 Hz, 1H, H_6_), 6.54 (d, *J* = 2.1 Hz, 1H, H_8_), 6.94 (d, *J* = 8.4 Hz, 1H, H_5′_), 7.64 (dd, *J* = 8.4, 2.1 Hz, 1H, H_6′_), 7.73 (d, *J* = 2.1 Hz, 1H, H_2′_), 8.69 (s, 1H, 3-OH), 9.61 (b, 1H, 4′-OH), 10.70 (b, 1H, 7-OH).

*Quercetin-5-OMe* (**Q6**). (50 mg, 0.16 mmol, 80%). ^1^H-NMR ([D6]DMSO, 300 MHz): 3.84 (s, 3H, 5-OCH_3_), 6.36 (d, *J *= 2.1 Hz, 1H, H_6_), 6.46 (d, *J *= 2.1 Hz, 1H, H_8_), 6.88 (d, *J* = 8.4 Hz, 1H, H_5′_), 7.49 (dd, *J *= 8.4, 2.1 Hz, 1H, H_6′_), 7.63 (d, *J* = 2.1 Hz, 1H, H_2′_), 8.62 (b, 1H, 3-OH), 9.30 (b, 1H, 3′-OH), 10.72 (b, 1H, 7-OH).

*Quercetin-7-OMe* (**Q7**). (55 mg, 0.17 mmol,85 %). ^1^H-NMR ([D6]DMSO, 300 MHz): 3.88 (s, 3H, 7-OCH_3_), 6.36 (d, *J *= 2.1 Hz, 1H, H_6_), 6.71 (d, *J* = 2.1 Hz, 1H, H_8_), 6.91 (d, *J* = 8.7 Hz, 1H, H_5’_), 7.59 (dd, *J* = 8.4, 2.1 Hz, 1H, H_6′_), 7.74 (d, *J* = 2.1 Hz, 1H, H_2′_), 9.49 (b, 1H, 3-OH), 12.50 (s, 1H, 5-OH).

*Quercetin-4′-OMe* (**Q8**). (60 mg, 0.19 mmol, 95%). ^1^H-NMR ([D6]DMSO, 300 MHz): 3.86 (s, 3H, 4′-OCH_3_), 6.20 (d, *J *= 1.8 Hz, 1H, H_6_), 6.43(d, *J* = 2.1 Hz, 1H, H_8_), 7.09 (d, *J *= 8.7 Hz, 1H, H_5’_), 7.66 (bm, 2H, H_2′_ & H_6′_), 9.34 (s, 1H, 3′-OH), 9.47 (b, 1H, 3-OH), 10.82 (b, 1H, 7-OH), 12.46 (s, 1H, 5-OH).

#### 4.3.18. Quercetin-5,7,3′,4′-OMe (**I3**)

*Quercetin-5,7,3′,4′-OMe* (**I3**) was available from an earlier study, and was obtained by the demethylation of **Q5** with an excess of AlCl_3_[21].

## 5. Conclusions

To chemically modify quercetin (**Q**) while retaining most of its antioxidant capacity, substitution at the 5 and 7 OH are the most suitable. This modification will not disturb the planar structure that enables the distribution of the electron donating effect through the π-system found in **Q**, and does not compromise H-bond interactions that can be essential for the stabilization of the flavone radical. This opens new avenues for (semi)-synthetic derivatives of **Q** that are chemically modified at the 5 or 7 position.
